# A Nanoformulation of Ubiquinol and Selenium Promotes Proliferation of Human Induced Pluripotent Stem Cells

**DOI:** 10.3390/antiox14091100

**Published:** 2025-09-10

**Authors:** Filomain Nguemo, Hai Zhang, Annette Koester, Susan Rohani, Sureshkumar Perumal Srinivasan, Jürgen Hescheler

**Affiliations:** 1 Centre for Physiology, Faculty of Medicine and University Hospital Cologne, University of Cologne, Robert-Koch-Str. 39, 50931 Cologne, Germanysureshps_bio@yahoo.com (S.P.S.); j.hescheler@uni-koeln.de (J.H.); 2 State Key Laboratory of Southwestern Chinese Medicine Resources, College of Pharmacy, Chengdu University of Traditional Chinese Medicine, Chengdu 611137, China; zhanghai@cdutcm.edu.cn

**Keywords:** QuinoMit Q10 fluid, hiPSCs, proliferation, mitochondria, cryopreservation

## Abstract

Human induced pluripotent stem cells (hiPSCs) hold immense promise for regenerative medicine. However, a critical barrier to the clinical application of hiPSCs is the difficulty in promoting robust cell proliferation while preserving their pluripotent state. Efficient hiPSC expansion without loss of pluripotency is crucial for generating high quality cells or therapeutic applications, disease modeling, and drug discovery. In our study, we investigated the effects of QuinoMit Q10^®^ fluid (QMF-Se), a nanoformulated supplement containing Ubiquinol (the active form of Coenzyme Q10) and Selenium, on hiPSC growth and maintenance in vitro. Interesting, QMF-Se supplementation significantly enhances hiPSC proliferation compared to control cultures. This increase in cell number was accompanied by heightened mitochondrial activity, suggesting improved cellular energy metabolism. Importantly, the expression of core pluripotency markers OCT4, NANOG, and SOX2 remained unaltered, confirming that the stem cells retained their undifferentiated status. Moreover, we observed that QMF-Se treatment conferred protective effects during the freeze–thaw process, reducing cell death and supporting post-thaw recovery. These results indicate that QMF-Se may improve both cell culture efficiency and cryopreservation outcomes. Overall, our findings highlight the potential of QMF-Se as a valuable additive for hiPSC culture systems, contributing to more efficient and reliable expansion protocols in regenerative medicine research.

## 1. Introduction

Human induced pluripotent stem cells (hiPSCs) have revolutionized regenerative medicine by offering an inexhaustible source of pluripotent cells capable of differentiating into any cell type of the human body [1]. This versatility makes them invaluable for applications ranging from disease modelling and drug screening to cell-based therapies. However, a significant challenge remains in maintaining hiPSCs in a highly proliferative yet pluripotent state during prolonged culture. Extended in vitro expansion often leads to stress-induced differentiation, metabolic dysregulation, and genomic instability, all of which can compromise their therapeutic potential.

Energy metabolism plays a pivotal role in sustaining hiPSC homeostasis [2]. Unlike somatic cells that predominantly rely on oxidative phosphorylation, hiPSCs exhibit a glycolytic phenotype, utilizing glycolysis as their primary energy source even in the presence of oxygen, a phenomenon known as the Warburg effect [3]. This metabolic profile supports their rapid proliferation and minimizes ROS generation [4,5]. Despite this glycolytic preference, mitochondria remain essential for maintaining cellular homeostasis, including the regulation of reactive oxygen species (ROS), Ca^2+^ signaling, and the synthesis of metabolic intermediates required for cell growth and epigenetic regulation [2,6]. Disruption of mitochondrial function can lead to elevated oxidative stress and impaired bioenergetics, both of which threaten the integrity and pluripotency of hiPSC cultures [7,8,9].

Coenzyme Q10 (CoQ10), a lipid-soluble component of the mitochondrial electron transport chain, plays a dual role as an electron carrier and a powerful antioxidant [10]. It has been shown to enhance mitochondrial efficiency and mitigate oxidative damage in various cell types, including stem cells [11,12]. These properties suggest that CoQ10 could support the metabolic demands of proliferating hiPSCs and protect against oxidative stress-induced differentiation [13]. Clinical trials have shown that CoQ10 supplementation led to a significant improvement in ejection fraction and quality of life [14].

Selenium is a vital trace element involved in the activity of selenoproteins such as glutathione peroxidases and thioredoxin reductases, which protect mitochondria from oxidative stress and support redox balance [15]. Deficiency in selenium has been linked to impaired mitochondrial function, while supplementation has been shown to preserve membrane potential, improve respiration, and reduce ROS production [16]. Clinically, selenium contributes to cardiovascular protection and may help counteract age-related mitochondrial decline [17].

However, the clinical and in vitro application of native CoQ10 is limited by its hydrophobic nature, which reduces solubility in aqueous culture systems and hampers cellular uptake [18]. To address these limitations, a nanoformulated version of reduced CoQ10 (Ubiquinol) combined with Selenium, marketed as QuinoMit Q10^®^ fluid (QMF-Se), has been developed to enhance bioavailability and mitochondrial delivery [17,19]. This cell culture media-soluble formulation offers improved cellular uptake, potentially allowing more effective modulation of mitochondrial function in hiPSCs.

In this study, we investigate whether or not QMF-Se supplementation can promote hiPSC proliferation without compromising their pluripotent characteristics. Specifically, we assess the impact of QMF-Se on cell growth kinetics, DNA synthesis, and the expression of core pluripotency markers, including OCT4, SOX2, and NANOG. Our findings suggest that QMF-Se represents a promising strategy for optimizing hiPSC culture by enhancing mitochondrial performance and cellular resilience to oxidative stress.

## 2. Materials and Methods

### 2.1. Chemicals and Reagents

Unless otherwise stated, all chemicals used for this study were purchased from Thermo Fisher (Waltham, MA, USA). Matrigel (354277) was purchased from Corning (Corning, NY 14831, USA).

### 2.2. Cell Cultures

Experiments were conducted using the IMR90 (hiPSCs) line, under authorization from the Robert Koch Institute (Berlin, Germany; license number: AZ 3.04.0210083). Cells were culture as previously described [20]. In brief, cells were maintained on Petri dishes coated with Matrigel and cultured in mTeSR™ iPS-Brew XF medium (Miltenyi Biotec, Bergisch Gladbach, Germany), supplemented with 50 U/mL penicillin and 50 U/mL streptomycin (Thermo Fisher Scientific, Waltham, MA, USA), at 37 °C in a 5% CO_2_ atmosphere. Once the culture reached approximately 90% confluency, the cells were detached using TrypLE (Thermo Fisher Scientific, Waltham, MA, USA). For passaging, cells were resuspended and cultured again in the same medium, now supplemented with 10 μM ROCK inhibitor.

### 2.3. Preparation of Treatment Compounds

QuinoMit Q10^®^ fluid + Selenium (QMF-Se), a nanoformulation of Ubiquinol and Selenium, was supplied by mse Pharmazeutika (Bad Homburg, Germany). Based on previous publications [13,17], the stock solution of QMF-Se was diluted at a ratio of 1:300 in the culture medium, resulting in a final concentration of 193 µM ubiquinol and 1.9 µM selenium in the cell culture environment. A Placebo (PLB) solution (mse Pharmazeutika, Bad Homburg, Germany) served as carrier control and was administered in the same way as QMF-Se. Both QMF-Se and PLB were stored at room temperature in the dark. Doxorubicin (DOXO) was freshly diluted in cell culture medium at 1 µM and served as an apoptosis-inducing agent.

### 2.4. Proliferation Assays and Cryopreservation

Human induced pluripotent stem cells (hiPSCs) were cultured on Matrigel-coated 6-well tissue culture plates in mTeSR™ iPS-Brew XF medium (Miltenyi Biotec, Bergisch Gladbach, Germany), either untreated (control) or supplemented with QMF-Se or PLB. After 2 days, when cells reached approximately 90% confluency, they were washed once with DPBS and dissociated into single cells using CTS™ TrypLE™ Select Enzyme (Thermo Fisher Scientific, Waltham, MA, USA). For PI staining, cell suspensions were collected in mTeSR™ Plus medium and centrifuged at 300× *g* for 4 min. The resulting cell pellet was washed with cold DPBS and resuspended in 400 µL of Annexin V-FITC binding buffer as previously described [21]. Cells were incubated at 4 °C for 15 min, protected from light. Following this, 10 µL of propidium iodide (PI) was added, and cells were stained for 5 min at 4 °C. Apoptotic cells were subsequently quantified using flow cytometry.

### 2.5. Pluripotency Assessment

Expression of pluripotency markers (OCT4, NANOG, and SOX2) was evaluated via the following:

(a) ***Immunofluorescence Staining for Pluripotency Markers***

To assess the expression of pluripotency markers, undifferentiated human induced pluripotent stem cells (hiPSCs) from the control, QMF-Se-treated, and PLB-treated groups were fixed with 4% paraformaldehyde, permeabilized with 0.1% Triton X-100, and blocked with 1% bovine serum albumin (BSA). Cells were incubated overnight at 4 °C with primary antibodies against SOX2, NANOG, and OCT4, followed by incubation with Alexa Fluor-conjugated secondary antibodies. Nuclei were counterstained with Hoechst 33342. After staining, samples were mounted using Invitrogen ProLong™ Gold Antifade Reagent (Thermo Fisher Scientific, Waltham, MA, USA) and imaged using an Axiovert fluorescence microscope (Carl Zeiss AG, Oberkochen, Germany) equipped with Axiovision 4.5 image analysis software.

(b) ***Quantitative PCR (qPCR) analysis***

For gene expression analysis, total RNA was extracted from cells using Invitrogen TRIzol™ Reagent (Thermo Fisher Scientific, Waltham, MA, USA), following the manufacturer’s instructions. Complementary DNA (cDNA) was synthesized as previously described [22]. Quantitative real-time PCR (qRT-PCR) was carried out using SYBR^®^ Green PCR Master Mix (Qiagen, Hilden, Germany) on a real-time PCR system. GAPDH was used as an internal reference gene for normalization, and relative gene expression levels were calculated using the ΔΔCt method. All primer sequences used in this study are listed in Table 1.

### 2.6. Assessment of Oxidative Stress and Cells Viability

To evaluate oxidative stress-induced cellular damage, human induced pluripotent stem cells (hiPSCs) were exposed to doxorubicin (DOXO), a known inducer of reactive oxygen species (ROS) [23,24]. Prior to DOXO treatment, a subset of cells was pre-treated without (control) or with QMF-Se or Placebo (PLB) to investigate the potential protective effects of QMF-Se against oxidative stress. Following treatment, cells were collected and analyzed using flow cytometry to assess cell viability as described elsewhere [25].

### 2.7. Mitochondrial Biogenesis Analysis

Mitochondrial biogenesis was evaluated using an ELISA-based assay MitoBiogenesis™ In-Cell ELISA Kit (Abcam, Cambridge, CB2 0AX, UK) according to manufacturer’s recommendation. hiPSCs were cultured under control, QMF-Se PLB, DOXO, or in combinations and seeded in 96-well plates following the staining protocol as described by Zimmerman MA et al. [26]. Cytochrome c oxidase subunit I (COX-I) levels were quantified by horseradish peroxidase (HRP)-mediated colorimetric detection, while succinate dehydrogenase complex flavoprotein subunit A (SDH-A) was measured using alkaline phosphatase (AP) development. The enzymatic reactions were developed in two sequential steps: first using an AP development solution, followed by an HRP development solution. Reactions were monitored for 15 min, with optical density (OD) readings recorded at 1 min intervals. Data analysis was performed as described [26,27].

### 2.8. Statistical Analysis

Statistical analyses were performed using Student’s t-test for comparisons between two groups and one-way analysis of variance (ANOVA) for comparisons involving multiple groups. A *p*-value of less than 0.05 was considered statistically significant. Unless otherwise specified, error bars represent the standard error of the mean (SEM). All analyses were conducted using SigmaPlot 14 and GraphPad Prism (Version 4.00 for Windows; GraphPad Software, San Diego, CA, USA).

## 3. Results

### 3.1. QMF-Se Modulates Colony Morphology and Enhances Proliferation of hiPSCs

**Figure 1:**
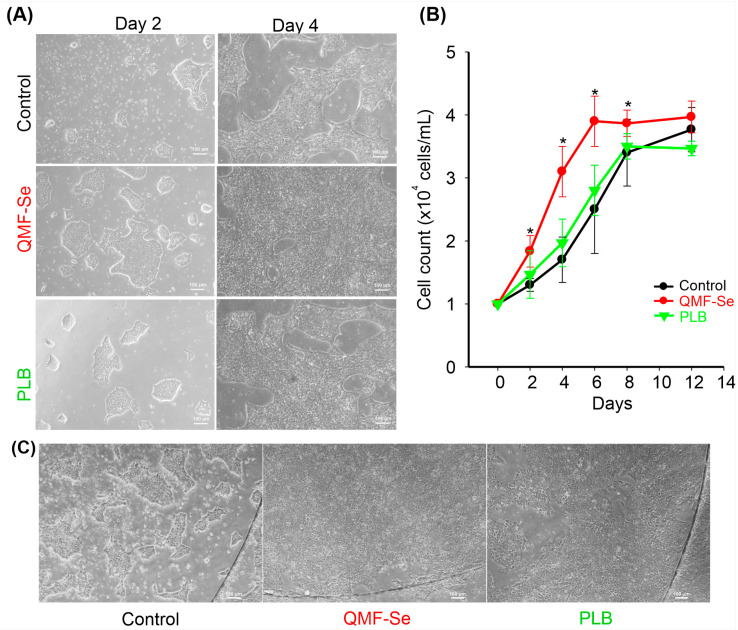
Assessment of QMF-Se effects on hiPSC colony morphology and proliferation. (**A**) Representative microscopy images of human induced pluripotent stem cells (hiPSCs) cultured on plastic dishes under control (PLB) and treatment (QMF-Se) conditions at day 2 and day 4 post-passage. Images illustrate differences in colony confluency, size, and morphology between conditions over time. (**B**) Quantification of cell number was performed using the EVE™ Automatic Cell Counter to assess cell proliferation. Data are presented as the total number of cells per sample at each time point. (**C**) Representative image of hiPSCs cultured on a glass coverslip, captured at day 2 post-passage, showcasing the colony morphology. * *p* < 0.05 vs. control. Scale bar: 100 μm.

Treatment with QMF-Se significantly affected both colony morphology and proliferation of human induced pluripotent stem cells (hiPSCs). Microscopy at days 2 and 4 post-passage revealed that QMF-Se-treated hiPSCs exhibited increased colony size, greater confluency, and more defined, compact morphology compared to the placebo (PLB) control (Figure 1A). These morphological improvements were evident from day 2 and became more pronounced by day 4. Quantification of total cell number using the EVE™ Automatic Cell Counter confirmed enhanced proliferation under QMF-Se treatment at both time points (Figure 1B). Furthermore, imaging of hiPSCs cultured on glass coverslips at day 2 post-passage demonstrated more robust and well-organized colony formation under QMF-Se treatment (Figure 1C). This observation not only highlights the proliferative effect of QMF-Se but also underscores the influence of surface quality on cell plasticity and colony architecture. The enhanced morphology on glass suggests that optimized surface conditions may synergize with treatment to promote stem cell health and maintenance in culture.

### 3.2. Protective Effect of QMF-Se on DOXO-Induced Cytotoxicity in hiPSCs

**Figure 2:**
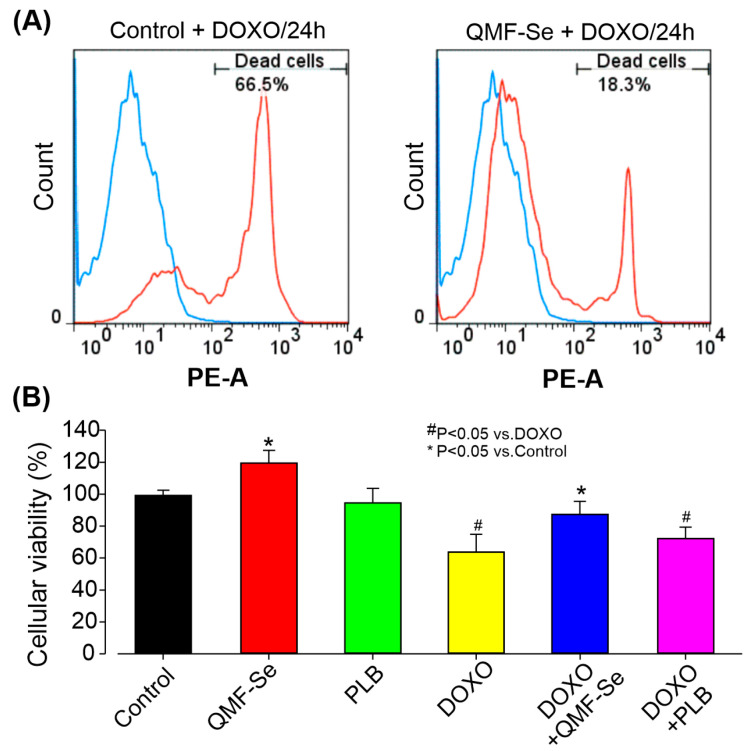
Protective effect of QMF-Se on DOXO-induced cytotoxicity in hiPSCs. (**A**) Representative flow cytometry plots illustrating the percentage of dead cells in hiPSCs treated with DOXO-treated for 2 h, with and without (Control) prior pre-treatment with QMF-Se for 24 h. Pre-treatment with QMF-Se significantly mitigated cell death induced by DOXO (300 nM), highlighting its potential as a protective agent against chemotherapeutic-induced cytotoxicity. (**B**) Quantitative summary of flow cytometry data, showing the protective effect of QMF-Se on cell viability. Results are expressed as mean ± SE; n = 3 independent biological replicates. * *p* < 0.05 vs. control, ^#^ *p* < 0.05 vs. DOXO.

Treatment with DOXO resulted in a significant increase in cell death among hiPSCs, indicating a pronounced cytotoxic effect. Flow cytometry analysis (Figure 2A) revealed a notably higher proportion of non-viable cells in the DOXO-treated group compared to the untreated control. However, pre-treatment with QMF-Se prior to DOXO exposure markedly reduced cell death and increased the proportion of viable cells relative to the DOXO-only group. These findings suggest that QMF-Se exerts a protective effect against DOXO-induced cytotoxicity in hiPSCs. The quantitative analysis of the flow cytometry data (Figure 2B) further supported this observation. In the DOXO-only group, cell viability dropped significantly, reflecting the sensitivity of hiPSCs to chemotherapeutic insult. The level of protection observed brought the percentage of viable cells closer to that of untreated controls, indicating that QMF-Se can effectively counteract the cytotoxic effects of DOXO. These findings, consistently observed across three independent biological replicates, reinforce the potential of QMF-Se as a cytoprotective agent. The ability of QMF-Se to preserve cell viability under chemotherapeutic stress may be of particular relevance in clinical or regenerative medicine contexts, where maintaining the integrity of pluripotent stem cell populations is critical.

### 3.3. QMF-Se Enhances Mitochondrial Biogenesis and Counters DOXO-Induced Mitochondrial Suppression

**Figure 3:**
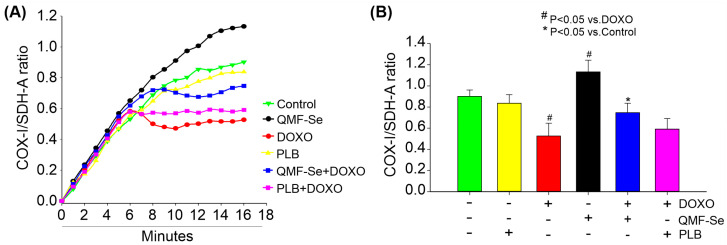
QMF-Se enhances mitochondrial biogenesis. (**A**) Detection of mitochondrial biogenesis using an ELISA-based assay following treatment with PLB (1:300), DOXO (300 nM), QMF-Se (1:300), or a combination. The optical density of COX-I was measured through horseradish peroxidase enzyme development, while Succinate dehydrogenase complex flavoprotein subunit A (SDH-A) was detected via alkaline phosphatase (AP) enzyme development for 15 min. The ratios shown in the graph represent optical densities recorded at 1 min intervals during each reaction. (**B**) Average ratio of mitochondrial DNA-encoded COX-I to nuclear DNA-encoded SDH-A at the 15 min endpoint for each treatment group. The COX-I/SDH-A ratio was calculated to assess the relative increase in mitochondrial DNA-encoded proteins, indicating enhanced mitochondrial biogenesis, as this was accompanied by no corresponding increase in nuclear DNA-encoded proteins. Results are expressed as mean ± SE; n = 3 independent biological replicates. * *p* < 0.05 vs. control, ^#^ *p* < 0.05 vs. DOXO.

QMF-Se treatment significantly enhanced mitochondrial biogenesis, as indicated by an elevated COX-I/SDH-A ratio (Figure 3A), a widely used marker of mitochondrial activity and replication. At the 15 min time point (Figure 3B), QMF-Se-treated cells showed a COX-I/SDH-A ratio of 1.13 ± 0.11, representing a 2.1-fold increase compared to the untreated control group (0.80 ± 0.06, *p* < 0.01) and more than a 3-fold increase relative to the DOXO-treated group (0.52 ± 0.12, *p* < 0.001). This robust increase suggests that QMF-Se rapidly activates mitochondrial biogenic pathways, likely through transcriptional or post-transcriptional mechanisms regulating mitochondrial enzymes.

In contrast, treatment with PLB alone resulted in a slight, non-significant reduction in the COX-I/SDH-A ratio (0.83 ± 0.08, *p* > 0.05 vs. control), indicating minimal influence on mitochondrial biogenesis under these conditions. Notably, co-treatment with QMF-Se and PLB did not produce a synergistic or additive effect attribute to QMF-Se. This outcome suggests that the biogenic effect is primarily attributable to QMF-Se alone. As expected, DOXO treatment significantly impaired mitochondrial biogenesis, as evidenced by a marked decrease in the COX-I/SDH-A ratio (0.53 ± 0.12, *p* < 0.05 vs. control) (Figure 3B), consistent with its known mitochondrial toxicity and disruption of electron transport chain function. Together, these results unveil QMF-Se as a dual-action modulator of mitochondrial health, capable of not only amplifying mitochondrial biogenesis under baseline conditions but also counteracting the deleterious effects of DOXO. This dynamic response positions QMF-Se as a promising therapeutic candidate with the unique ability to preserve and restore mitochondrial function under chemotherapeutic stress, potentially opening new avenues for mitochondrial-targeted cytoprotection in pathological conditions.

### 3.4. QMF-Se Treatment Maintains Pluripotency Marker Expression and Modulates Epigenetic Regulator Levels in hiPSCs

**Figure 4:**
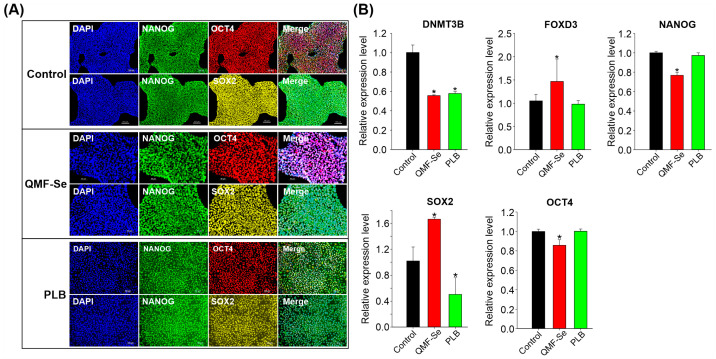
Pluripotency marker expression and epigenetic regulation in control and QMF-Se-treated hiPSCs. (**A**) Representative immunofluorescence images showing the expression of key pluripotency markers OCT4, NANOG, and SOX2 in hiPSCs from three groups: untreated control, QMF-Se-treated, and PLB-treated. Cells were stained with specific antibodies against each marker, and nuclei were counterstained with Hoescht (blue). Scale bar: 100 µm. (**B**) Quantitative RT-PCR analysis of the expression levels of DNMT3B, a de novo DNA methyltransferase involved in epigenetic regulation, along with core pluripotency genes OCT4, NANOG, SOX2 and FOXD3. mRNA levels were compared among control, QMF-Se-, and PLB-treated hiPSC groups. GAPDH was used as an internal control. Results are expressed as mean ± SE; n = 3 independent biological replicates. * *p* < 0.05 vs. control.

Immunofluorescence imaging revealed that hiPSCs from the untreated control, QMF-Se-treated, and PLB-treated groups consistently expressed the core pluripotency markers OCT4, NANOG, and SOX2 at high levels (Figure 4A). These markers displayed strong nuclear localization, which is characteristic of their active roles in maintaining pluripotency, and this pattern was preserved across all treatment conditions. The preservation of both expression intensity and cellular localization strongly suggests that treatment with QMF-Se does not impair the presence or functional positioning of these critical transcription factors within the cells.

To complement the protein-level observations, quantitative RT-PCR analysis was performed to assess the transcriptional expression of those key pluripotency genes. The analysis confirmed that OCT4, NANOG, and SOX2 mRNA transcripts were present in all experimental groups (Figure 4B), indicating that the core pluripotency gene network remains active following treatment. However, a more nuanced expression profile emerged in the QMF-Se-treated group: SOX2 and FOXD3 mRNA levels were significantly upregulated compared to both the untreated control and PLB-treated groups. FOXD3 is known for its role in pluripotency maintenance and early lineage decisions, so its elevation may suggest enhanced regulatory activity in these cells. Conversely, NANOG and OCT4 transcripts were found to be downregulated in the QMF-Se group relative to controls, which may indicate a shift in the balance of pluripotency factors or a subtle modulation of the pluripotent state. In addition to key transcription factors, the de novo DNA methyltransferase DNMT3B exhibited altered expression in response to both QMF-Se and PLB treatments. These changes suggest that the compounds may influence regulatory mechanisms at the epigenetic level, contributing to the observed transcriptional shifts. Notably, this modulation occurred without disrupting the pluripotent identity of the hiPSCs, indicating a selective and controlled adjustment of cellular programs.

Taken together, QMF-Se preserves core pluripotency in hiPSCs while selectively modulating key factors, potentially through epigenetic regulation, without altering the cells’ fundamental identity.

### 3.5. QMF-Se Treatment Preserves hiPSC Viability Following Cryopreservation

**Figure 5:**
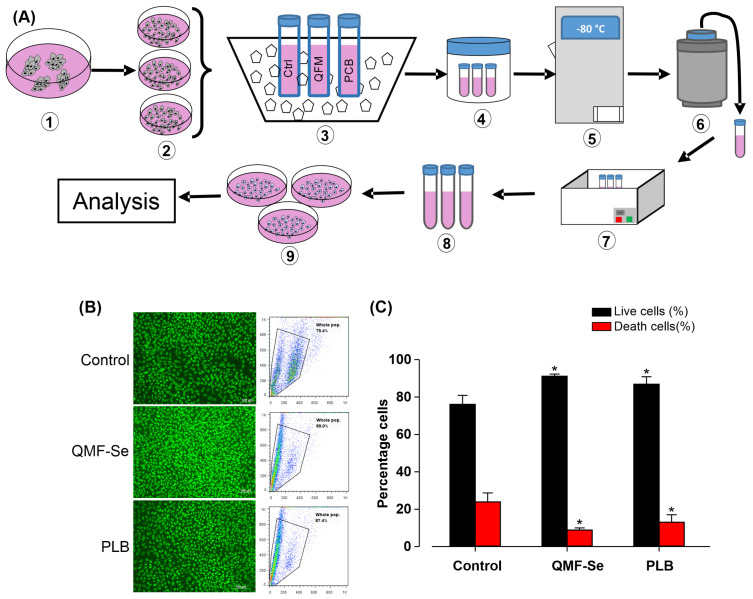
QMF-Se does not affect the cryopreservation of hiPSCs. Human induced pluripotent stem cells (hiPSCs) were thawed directly from cryopreserved samples. Cells were either analyzed immediately by flow cytometry or fixed and stained with propidium iodide (PI) and FITC-dUTP, then evaluated by fluorescence imaging. The percentages of live and dead cells were assessed. (**A**) Schematic overview of the main steps involved in the freezing and thawing process of hiPSCs. (**B**) Representative fluorescence image (left) and flow cytometry analysis (right) of viable hiPSCs. (**C**) Quantification of the percentage of live and dead cells in control, QMF-Se, and PLB-treated groups. Data are presented as mean ± standard error (SE); n = 3 independent biological replicates. * *p* < 0.05 vs. control.

To evaluate the protective effect of QMF-Se on human induced pluripotent stem cells (hiPSCs) during cryopreservation, cells were subjected to a standardized freeze–thaw protocol (Figure 5A) designed to minimize variability across samples. Immediately upon thawing, cell viability was assessed using two complementary methods: fluorescence imaging following staining with propidium iodide (PI) and FITC-dUTP to label dead and apoptotic cells, respectively, and flow cytometric analysis to quantify live versus dead populations. Representative fluorescence images (Figure 5B, left) revealed that QMF-Se-treated hiPSCs exhibited fewer PI-positive (dead) cells and reduced FITC-dUTP incorporation compared to control, indicating lower apoptosis and membrane compromise. Flow cytometry profiles (Figure 5B, right) corroborated these observations, showing a higher proportion of viable cells in the QMF-Se group relative to both control and PLB-treated cells. Quantitative analysis of viability (Figure 5C) demonstrated a statistically significant increase in the percentage of live cells in the QMF-Se treatment group, with viability rates of approximately 91.1 ± 1.2% compared to 76.1 ± 4.8% in controls and 86.9 ± 4.0% in PLB-treated samples (*p* < 0.05). This suggests that QMF-Se confers enhanced cellular protection during the freeze–thaw process, potentially by stabilizing cellular membranes or mitigating apoptosis pathways. Altogether, these results confirm that QMF-Se is compatible with standard cryopreservation protocols and significantly improves hiPSCs survival post-thaw. This property makes QMF-Se a promising additive for stem cell banking and therapeutic applications where maintaining high cell viability after storage is critical.

## 4. Discussion

This study demonstrates that QMF-Se enhances multiple aspects of human induced pluripotent stem cell (hiPSC) condition, such as proliferation, mitochondrial activity, and maintenance of pluripotency, through coordinated transcriptional and metabolic regulation. QMF-Se treatment led to early and sustained improvements in cell colony and morphology, higher confluency, and more compact organization. These changes were accompanied by significant increases in total cell number, suggesting that QMF-Se may at least in part enhance proliferative capacity without inducing spontaneous differentiation. Importantly, QMF-Se preserved expression of the core pluripotency transcription factors OCT4, NANOG, and SOX2 at both the protein and transcript levels. Although OCT4 and NANOG transcripts were moderately downregulated, their expression and localization remained intact, suggesting that post-transcriptional mechanisms may help sustain functional levels. It is also worth noting that the downregulation or loss of these factors can play an active role in initiating differentiation and germ layer specification. Interestingly, SOX2 expression was significantly upregulated following QMF-Se treatment. SOX2 plays a central role in maintaining self-renewal and preventing differentiation and was shown to be differently regulated from NANOG and OCT4 in human embryonic stem cells [28]. Its elevation in hiPSCs may reflect a reinforcement of pluripotency in response to QMF-Se. Additionally, the upregulation of FOXD3, a transcription factor involved in safeguarding pluripotency and modulating early lineage decisions, suggests a transcriptional landscape that promotes stem cell identity while enhancing cellular resilience [29]. In contrast, the observed changes in DNMT3B expression suggest a potential epigenetic mechanism underlying these effects. As a de novo DNA methyltransferase, DNMT3B plays a critical role in establishing methylation patterns that regulate gene expression in early development and the maintenance of pluripotency [30]. Its modulation by QMF-Se may at least in part indicate subtle epigenetic remodeling that helps stabilize gene expression profiles supportive of long-term self-renewal.

Mitochondrial biogenesis relies on the finely tuned and coordinated expression of genes from both mitochondrial DNA (mtDNA), such as cytochrome c oxidase subunit I (COX I), and nuclear DNA (nDNA), such as succinate dehydrogenase subunit A (SDH-A) [31,32], which together produce the essential components of the oxidative phosphorylation (OXPHOS) machinery [33,34]. Some studies have investigated how cells maintain ideal balance between COX and SDH subunits in response to different environmental and developmental signals using various tissues and cell lines [35,36,37]. In our study, QMF-Se significantly enhanced mitochondrial biogenesis, as evidenced by a marked increase in the COX-I/SDH-A ratio. This shift indicates a selective upregulation of mitochondrial DNA-encoded proteins, independent of nuclear-encoded mitochondrial components, and suggests activation of mitochondrial replication and transcription. Enhanced mitochondrial output is often correlated with increased energy demands during proliferation or metabolic shifts toward oxidative phosphorylation [38]. In pluripotent cells, which typically rely on glycolysis, this increase in mitochondrial activity may indicate a more metabolically flexible or energetically balanced state that supports high proliferation without compromising pluripotency. Given the observed changes in mitochondrial function alongside the maintenance of pluripotency markers, it is likely that QMF-Se enhances energy metabolism in a way that sustains stemness rather than promoting spontaneous differentiation.

Moreover, QMF-Se conferred protection against mitochondrial and cellular stress induced by doxorubicin (DOXO), a chemotherapeutic agent known to cause oxidative damage and impair mitochondrial function. QMF-Se pre-treatment preserved mitochondrial biogenesis and significantly reduced DOXO-induced cell death, suggesting a cytoprotective role that may involve preservation of mitochondrial integrity or activation of stress response pathways [39]. By maintaining mitochondrial function under stress and enhancing basal mitochondrial biogenesis, our nanoformulated QMF-Se may improve ATP production, reduce the buildup of reactive oxygen species (ROS), and support energy-dependent processes that are vital for stem cell survival and growth. CoQ10 has been shown to play a key role in mitochondrial energy production by shuttling electrons between complexes I and II to complex III in the electron transport chain during oxidative phosphorylation, a crucial step in ATP synthesis [40]. Beyond its role in energy metabolism, CoQ10 functions as a vital lipid-soluble antioxidant, helping to shield mitochondria from damage caused by reactive oxygen and nitrogen species produced during respiration. Additionally, CoQ10 is involved in various other mitochondrial processes such as pyrimidine and fatty acid metabolism, the activity of uncoupling proteins, and the regulation of mitochondrial permeability transition pores [37,41,42].

## 5. Conclusions

Taken together, these findings suggest that QMF-Se supports a highly favorable cellular environment for hiPSC maintenance by reinforcing pluripotency networks (via SOX2 and FOXD3), subtly modulating epigenetic regulators (through DNMT3B), and promoting mitochondrial biogenesis and energetic efficiency. This integrated transcriptional and metabolic response likely underpins the observed improvements in cell viability, proliferation, and cryopreservation recovery. These properties make QMF-Se a promising additive for stem cell culture and preservation, with potential applications in regenerative medicine, disease modelling, and stem cell-based therapies.

## Figures and Tables

**Table 1:** List of genes and primers used for qRT-PCR.

Gene Names	Sequence (5’ to 3’)	Amplicon Size (bp)	Annealing Temp. (°C)
**DNMT3B**	**F**: GTCGTGCAGGCAGTAGGAAA **R**: GCCATTTGTTCTCGGCTCTG	175	60
**FOXD3**	**F**: GCAACTACTGGACCCTGGAC **R**: CTGTAAGCGCCGAAGCTCT	145	60
**NANOG**	**F**: ACTAACATGAGTGTGGATCC **R**: TCATCTTCACACGTCTTCAG	130	60
**SOX**	**F**: ATGCACCGCTACGACGTGA **R**: CTTTTGCACCCCTCCCATTT	437	60
**OCT4**	**F**:AGGGCAAGCGATCAAGCA **R**: GGAAAGGGACCGAGGAGTA	168	60
**GAPDH**	**F:** CAAGAGCACAAGAGGAAGAGAG **R:** CTACATGGCAACTGTGAGGAG	102	60

## Data Availability

The datasets generated during and/or analyzed during the current study are available from the corresponding author on reasonable request.

## Abbreviations

The following abbreviations are used in this manuscript:

AP	alkaline phosphatase
BSA	bovine serum albumin
CoQ10	Coenzyme Q10
COX-I	Cytochrome c oxidase subunit I
DOXO	doxorubicin
hiPSCs	human induced pluripotent stem cells
PLB	placebo
QMF-Se	QuinoMit Q10^®^ fluid + Selemiun
ROS	reactive oxygen species
SDH-A	succinate dehydrogenase complex flavoprotein subunit A
